# A Plea for the Adoption of Oral Prophylaxis

**Published:** 1906-12-15

**Authors:** Grace P. Rogers


### A PLEA FOR THE ADOPTION OF ORAL PROPHYLAXIS.

BY GRACE P. ROGERS, D. D. S.,

Read before the Detroit Dental Society, May 10, 1906.

A little more than half a century ago the subject of oral or dental hygiene was first considered by our profession. Every year since then a few men have tried to arouse interest in this all-important topic, but their efforts seemed to produce only temporary enthusiasm, which, however, was not in vain. The advocates were sincere, and even though they seemed to stand alone, did not despair, but continued to study the subject.

Progress of a profession is the result of thought, effort, and time spent in some direction by earnest men, and as a result of such an effort we have a new branch in our profession called Oral Prophylaxis. The advocates of oral hygiene laid a firm foundation, upon which Dr. D. D. Smith has built so well the principles and technique of Oral Prophylaxis.

These two subject have so much in common that we seldom see any distinction made between the two. Yet I believe there are differences which we should recognize. For example: a man may take his daily baths and exercise, select his food carefully and eat it properly, surround himself with plenty of fresh air, never worry, drink all the pure water necessary, and, in fact do everything he can to preserve his health and prevent disease; this man is living according to the principles of hygiene. However if to his own care he add that of his physician, who, after putting the patient's system in a normal condition, will make regular examinations of the fluids and organs of the body, thus being able to anticipate and ward off disease, then he is carrying out the ideas of Prophylaxis.

Our patients may be faithful in the use of tooth-brushes, tooth-powder, dental floss and mouth washes, and to the best of their ability correct abnormal conditions and prevent disease in the oral cavity, yet this is not oral prophylaxis, but oral hygiene. On the other hand, if we place those mouths in an absolutely healthy state, and then, by regular treatments, stimulate and increase the resistance of the mouth tissues, direct these patients in their personal care, anticipate and prevent disease, control conditions absolutely, then we are practicing Oral Prophylaxis.

In oral hygiene, it is necessary for the dentist to do much in the way of removing accumulations which a patient under prophylaxis treatment does for himself. In oral hygiene the care of the mouth is an unpleasant task for both patient and dentist, while in oral prophylaxis it is a pleasure. The former prevents some disease, but the latter prevents more.

A few years ago oral prophylaxis was spoken of as a treatment which would cure pyorrhea and prevent some decay. Now it is a specialty in dentistry, not for dental nurses, but for graduate dentists. It is that branch of dentistry which has for its object the restoring of the mouth tissues to a state health and preserving them in this condition by increasing their resistance to hurtful influences, and by anticipating and removing the cause of disease in them. According to this definition we have included more than the name implies, for the meaning of the phrase is the prevention of disease in the oral cavity by manipulative interference.

No mouth is entirely healthy which has not been under this treatment, therefore we must precede our preventive treatment by a curative one.

To those who have not given attention or thought to this work, oral prophylaxis suggest tooth-cleaning and the monotonous task of polishing teeth once a month, which seems nonsensical and unworthy of a professional man's time. Tooth cleaning is indeed unworthy of our time unless it is to be followed by regular treatment. The first time a patient presents himself for the work, it is always necessary to clean out the oral cavity, and until he has learned to do it for himself, we must help him. As soon as we find he is able to give his mouth the proper care, we have the right to reprove if our instructions have been neglected. It may take some time for him to learn to do his part as it is not an easy lesson, but we must keep up his courage and our own patience.

We should drop the phrase "tooth-cleaning" from our vocabulary and educate our patient to do the same. If a woman were to go to a hair store and tell the person in charge that she wanted her hair cleaned, she would be met with a cold look of disapproval. If you are in doubt also ask your manicure to clean your nails some time, and watch the effect.

Why, should Mrs. Smith ask for a shampoo or a manicure and on her way home drop into her dentist's office and ask for an appointment to have her teeth cleaned? Who is responsible for this condition of affairs?

People have been educated in other lines, but not in dentistry, and until they are educated in refinement of expression our services will not be appreciated.

It rests with ourselves as to how soon a change will be made. It is our own fault that we are asked to clean teeth.

No dentist should ever perform this work as is unually done in nearly every dental office in the country. Every time we do it, we are lessening our self-respect, lowering ourselves in the estimation of our patients and our profession in the eyes of the world. I hear some one say, "We are doing our patients a good service." Yes it maybe a good service, but so insufficient. It is true that we relieve their mouths of large quantities of irritating and infectious material, but it will be but a short time before there will be a similar amount of deposit in that mouth. The tooth surfaces are not smooth and the roughened enamel tends to catch and hold coatings, food particles, germs and stains. The patient does not know and is not instructed how to care for his mouth.

The task is often a disagreeable one, both for operator and patient, and when quickly and perfuntorily done of no value.

Every dentist should take a decided stand and positively refuse to clean any patient's teeth unless he can follow up the work by systematic prophylaxis treatment until he has placed the mouth in a healthy condition. It is as much our duty to do this as it would be to refuse to only partially remove the decay in a cavity before filling. Patients must be impressed with the importance of the work. They must be taught the difference between this work and that to which they have been accustomed. We dare not ease our conscience by say-our patients do not want the treatment. As soon as they know what it is and have experienced the difference between a healthy and an unhealthy mouth, they will want it.

Oral Prophylaxis is a broad subject, and to simplify it, let us consider its different phrases separately. There are three, all of which are equally important and without any one of which you will not meet with success: (1) Instructive, (2) Technical, and (3) Executive

With instruction—Let us consider what is necessary when a case is first presented to us. We will take for example, a mouth such as the general practitioners sees in his office every day and to which he pays no attention after he has filled all cavities and made any other necessary repairs. The gums show a state of marked hyperemia, and have receded in se-eral places. Where there is recession, the teeth are sensitive at the necks. There is tarter, both salivary and serumnal, also much soft accumulation and many stains. The patient is usually dismissed without any attention being paid to this condition, which is a serious one. It may be that the dentist is not able to recognize the difference between this mouth and a really healthy one, because he may never have seen the latter. If he is able to make the distinction, he probably does not wish to be bothered with its treatment as he is too busy with other work, or is afraid he will not be able to get a good fee for his services. One writer has said, "There is no service so little appreciated by our patients and of so little remuneration to us as the care and attention we bestow in the treatment of the diseases affecting the gums. So much so is this the case and so ignorant do we find our otherwise intelligent patients in regard to these conditions that that many of us give only casual care, knowing if we go further that our object will be misconstrued and our solicitude will be taken for a desire to collect a fee''

Our patients *are* ignorant in this regards because *we* have not taken the trouble to instruct them, and there is no one else of whom they can learn if they would. Who taught them that cavities should be filled, or that an aching tooth could be relieved? No one, I am sure, but members of our profession. No one has told them that it is unnatural for gums to bleed or that pyorrheea can be cured. In fact most dentists give their patients a mouth-wash to stop bleeding of the gums (without removing the cause of the trouble), and tell them that there is no cure for pyorrhea, that sooner or later they must lose their natural teeth and have artificial substitutes.

This spring, as usual, hundreds of men will be graduated from the dental colleges of our country without any knowledge or experience in the treatment of diseased conditions of the gums or peridental membranes. The new graduates may be very keen in finding cavities and root canals, but they are totally blind to the condition of the soft tissues. They are taught to take care of the house and to let the foundation take care of itself. The result of all this is that we support specialists whose work is to extract teeth. Never in the history of dentistry was there anything which reflected so much disgrace upon us as this fact. It is time to awaken to the importance of saving the human teeth, not by filling alone but by keeping the soft tissues in a healthy condition.

But let us go back to our patient. He does not know his mouth is not healthy. He may have noticed the gum recession, the sensitive teeth probably annoy him, yet he cannot appreciate that this sensation is not due to caries. Unless you question him he will say nothing about his gums bleeding either because he thinks it natural, or he has become so accustomed to it that he does not notice it.

We should never begin operations without having made a detailed record of each case. Neither should we make one move toward treatment until we have with hand and mouth mirrors, shown the patient the condition of his mouth. First call his attention to the color of his gums. Find, if you can, a pink area, and with this compare a deep red, perhaps a purple area, and then point out and explain the cause. Point out places where the gums have receded and tell him why they have done so. Instruct him thoroughly as regards all the conditions present, and the results of these harmful influences, unless you can have control of his mouth. Now, to many of you this may seem a waste of time involving too much detail, but you will be rewarded amply if you will only take the trouble. If you simply tell your patient that he needs treatment and begin to work at once, he will wonder what you are doing, and, while he cannot help but realize the change after several treatments, he will not fully appreciate one-half that you have done for him, and may question the value of time spent in this way.

If such a patient has been under your care for a number of years it is your fault that his mouth is in this state, just as much as re-decay under one of your fillings would be your fault. So begin at once and try to make amends for this neglect, by restoring the unhealthy tissues to health. Patients must be taught how to brush their teeth, the exact number of times each day, when, where, and why. Give them a practical demonstration in the method of using the brush, powder and floss. The thorough mastication of food is important and you must impress your patients with this fact. Intelligent patients will be interested in the process of digestion, the cause of decay, any phase of bacteriology, anatomy, and physiology, all of which have a certain bearing upon this work. We need not stop working to impart knowledge, but should cultivate the habit of talking and educating our patients while working. You will raise yourself in their estimation if you show them that you know some thing more than the mechanical principles involved in filling teeth. You will of course, find some who are not interested, but for those few do not neglect to instruct those who are interested.

*Technical*—There are few operations in surgery more delicate or which require more skill than the scaling in pyorrhea pockets, and you will be called upon to treat many cases of that much-dreaded disease. It is only by developing the sense of touch to a high degree that we can hope to succeed in oral prophylaxis. You will need patience, for perhaps after working for one hour or more over one tooth you will think of what you might have accomplished in that same length of time if you had been working at filling. I might have something to show for my work, you say. But think one moment—are you not giving your patient a better service by saving this one tooth than if you placed a six-tooth bridge in his mouth in its unhealthy condition? The latter would last but a very short time, and by its holding deposits and soft accumulations would really hasten the loss of the abutments. Your patients come to you for your very best service, and you should give it to them regardless of your preference for certain kinds of work. As regards thoroughness, you must be conscientiously thorough. Unless you are, you will fail to obtain good results, which will discourage you in your efforts, as well as lessen the patient's faith in you and in the treatment. The patient must be thorough in his personal care, or improvement will be slow. If you ever find any soft deposits, even on the distal surface of a third molar, you should insist upon better care of the mouth. The thorough sterilization of instruments used cannot be too strongly emphasized.

For in the first treatments you are dealing almost entirely with lacerated and diseased soft tissues, and there is much danger of infection unless due precaution is taken.

*Executive*—In order to carry oral prophylaxis to a successful issue, it must be put on a systematic basis, and this demands the third phase of our subject, executive ability.

You must not let your patients dictate to you and tell you how often they want treatments, for you must govern and control everything absolutely. The true professional man understands his subject better than the layman, and this fact is the reason for the public's wishing to employ his skill. How can he give them his best service if he allows his judgment to be swayed by their ignorant notion of what should be done? A professional man should be an absolute monarch in his office. See to it that every patient is well equipped with everything that is necessary to obtain the results, viz: tooth-brushes, powder, dental floss, and a good mouth wash. Do not allow the patient to select any of these but insist that he use what you prescribe. A record should be kept of every case under treatment, and a note of conditions made after each treatment.

If you are a specialist in dentistry, oral prophylaxis expects much from you, but if you are in general practice, she demands more from you. To say that we cannot give up our "valuable time" to prophylaxis is only another way of saying that the prevention of disease is unworthy of our time.

If the regular practicing dentist has too much work to do the admittedly important work of preventing tooth destruction, would it not be better to have a few patients cared for in every way, with healthy mouths and teeth that are brilliantly polished, or would you have many patients whose teeth are constantly needing repairs, with inflamed tissues and mouths full of infection?

Some claim that few men like the work. They do not like it because they do not know anything about it. Nothing will more quickly or surely raise your ideals or inspire you with a desire to do better work than oral prophylaxis.

It has been said that tooth-polishing is a tedious, irksome, monotonous task. This is not true if the operator can see beyond the end of his stick. If he will only think of the far-reaching results of his work, he will enjoy every stroke he makes. There is stimulation being given the gums, the teeth, the peridental membrane. Health is being imparted to all the mouth tissues, and in many cases the general health is being improved. Also, mouth diseases of local origin are being prevented, and the gratitude of the patient is no minor consideration, for you are rendering him a service with which no other can compare.

If we would give our patients our very best services, shall we close our eyes to our duty so that we may not see the wholesale destruction of the peridental membrane and tooth tissue going on, because we would rather be making inlays or removable bridges? You may make if you please a porcelain inlay. It may be perfect in adaptation, contour, and color. Yet what have you? You have a repair which, no matter how good, is never as good as nature gave. Would you not be giving mankind a nobler and greater service if you were spending your time in studying the causes which produced the need for repair, and in so far as you were able, were preventing these conditions?

It is your duty to at least make a study of oral prophylaxis if you are in doubt about its importance, and do not think that because ignorance is bliss, it would be folly to be wise.

Exchange treaments with another dentist until you both have healthy mouths. You will be so pleased and grateful that you will put some patients on regular treatment at once. But do not take up the work unless you are going to do it as you should, and take it up in earnest. It is a great work, and we do not want dental nurses or unprofessional men trying to temporize with it. If you cannot take it up whole-heartedly, let it alone entirely and confess that you know nothing about it. Do not try to cover your ignorance on the subject by talking against it and thus retarding the efforts of those who are trying to advance it.

Some one has said, "The history of any country is the biography of its great men" and so the history of dentistry is the biography of its great men. If we could have a biograghy of Dr. D. D. Smith's life we might understand why dentistry is taking this vast stride forward. His life since he began the work has been one of entire self-sacrifice to his patients and to his profession. We owe him a debt of gratitude which can only be paid by our untiring efforts to advance this great work.

A very prominent man in the profession says he can make more money doing other work, and therefore does not intend to give his time to oral prophylaxis. He says he is in the profession for the money that is in it. It is decidedly unprofessional to take that attitude; we are not in a business, but in a profession, and we should not forget this: "A professional man is one who gives his life to the pursuit of his art or calling with unselfish devotion to those whom he is to serve, using every means at his command to perfect himself, both technically and scientifically. Under this definition few qualify, but all can who will."

The rank of dentistry among the professions depends upon the advance of oral prophylaxis. Are you satisfied with her present rank? If not, "put your shoulder to the wheel" and let us all move forward.

DISCUSSION.

Dr. W. H. Graham,: I never could quite understand the difference between oral hygiene and oral prophylaxis. If there is a difference, I think it is rather imaginary. It is like the difference given us, when we went to college, between infection and contagion, and between disinfectant and antiseptic.

I will confine myself to that phase of the paper which deals with pyorrhea alveolaris, for the reason that I know a little about it, and the discussion that I provoke may be of interest to us.

The essayist would have us conclude that oral hygiene and the real treatment of pyorrhea alveolaris is of very recent origin. It is, undoubtedly, getting more attention now than heretofore, but I question if the real treatment of pyorrhea alveolaris has advanced much in the last twenty years, except in the persistency with which it is treated. I read yesterday an extract from Dr. Garretson's book which contained an article by Dr. Allen written thirty-two years ago, and I will quote therefrom a few of his phrases.

He describes a pocket which he found on the lingual face of the central extending near ly to the apex of the root, also a pocket on the right central extending quite to the apex, also a left lateral so badly diseased that the nerve had died.

He then goes on to describe the condition in which he found those teeth and his treatment. He says he found that the trouble was due to an accumulation of dark tartar. With the scalers he removed that as perfectly as he could, and after several treatments with the scalers, he then treated it once or twice a week for eight weeks with peroxide of hydrogen and other mild antiseptics. He found at the end of three months those teeth had tightened almost perfectly. The elongated lateral had become firm and the pus was entirely stopped. The tissues had regained their normal condition and the mouth was in a healthy condition.

This was thirty-two years ago, so that when I say that the pyorrhea alveolaris treatment has not improved to the extent that we might expect I think I am giving the evidence for what I say. It is true that we now give more attention to the treatment. In the same article he adds, that had this tartar been discovered and removed the bad condition of these teeth would never have been. In other words, he also suggested the prophylaxis treatment.

Pyorrhea alveolaris, I think is the most difficult disease we have to deal with, and it is also the most discouraging.

Patients come to our offices and complain bitterly that their teeth have been neglected by their former dentist, and they plead with us to do what we can for them. We may be as persistent and careful, and as conscientious as we can, but if these teeth are badly affected we know that we cannot restore them to the normal condition. If the isolated tooth is suffering not too severely we know that we can by anchorage and the prophylaxis treatment that has been suggested cacomplish something; but if the arch is entirely involved and the pus is oozing from every socket, and the pockets are so extensive and deep that they reach almost to the apex of the roots, it is a safe diagnosis that all such teeth are practically beyond saving.

When our patients ask us as to the cause of this trouble, we offer all kinds of suggestions. The most prevalent suggestion given is a uric acid diathesis. I think this is a mistake, because the uric acid diathesis theory is becoming gradually exploded. It is also a serious mistake for us to say this if we do not know that the patient is actually suffering from such a condition. I save a good deal of time and trouble by telling patients that I do not know, but if their teeth are cared for in time and they get the proper treatment, much can be done for them.

With the advent of much discussion of this subject we have also in our dental journals much literature from the different pharmaceutical firms lauding their medicines as uric acid solvents, etc. I think we should be very careful in our employment of all such remedies. For the last two years the editor of the Journal of the American Medical Association has been giving us distinguished articles on what he calls the "Truth and Poetry of Uric Acid." It is very interesting, but not very instructive. After reading it, you will come to the conclusion that the Episcopalian did after reading the Westminister confession of faith. He said, after reaidng it "I'll be d—d if I will, and I'll be d—d if I won't."

We have coming to our desks a monthly journal called the Uric Acid Monthly; and we have there, of course nicely written articles and recommendations of the wonderful solvent action of special preparations, but I think it may be dangerous and surely an unwise thing for us to recommend any such remedies. I believe that in only a few cases the local or direct treatment will be most valuable. We have a few cases which are caused by malaria, some by drugs, and a few by other diseases. Perhaps in ninty percent. of all conditions, local treatment will prevail if taken in time.

It has been my custom for the last two years to prescribe drinking large quantities of water. I, myself, drink nearly ten glasses a day. When you tell your patients to drink more, don't make the mistake I did—Advising a friend of mine that he was not drinking enough, he went home one day with a "*load*" that he should have made two trips with, and he was upbraided by his wife, but he replied that I had told him he was not drinking half enough. So when you advise your patients to drink more, tell them to drink more "*water*".

Dr. G. P. Wood: I have been very much edified and pleased with many strong suggestions made in this paper. I have congratulated Dr. Rogers once before, and am glad to again give additional thanks for so helpful a presentation of this matter. Lest I appear to be over-enthusiastic along these lines, I tell you before I start that I have just made a visit to Dr. D. D. Smith, the apostle of Prophyalxis. There are others in this room who did the same thing, and, I speak of it now so that you will not think I am overly enthusiastic, and I do not want to spoil the impression this paper has made by my enthusiasm.

I think that every word of Dr. Rogers paper can be proven by the facts of these conditions when they become known.

I have been working along the lines that Dr. Rogers has developed this evening. I have been cleaning teeth, as we all have for a good many years, and I agree with the essayist heartily in the idea that we should at once blot out that term, we should stop the talk about cleaning teeth: call it "manicure" or anything else but never cleaning teeth.

Dr. Graham has spoken of this idea of curing pyorrhea and the treatment of it being so old. I believe it is commonly accepted that some people did cure pyorrhea even as far back as 1832, but cases were so rarely cured that it did not do people very much good, many were not even benefitted

The idea of oral prophylaxis and the treatment of pyorrhea alveolaris is on the verge of becoming an established specialty in dentistry and may be taken up by specialists all over the country. Where there were, two or three years ago, only two or three treating it with no success, there are now a good many succeeding and the number will be multiplied very fast in the near future. The enthusiasm and knowledge which has inspired this paper cannot be measured in dollars, nor estimated in its therapeutic values, by the great majority of practitioners. It is immensely valuable to the profession today in this part of the country, as we shall know more of its results than in any other section, because we have Dr. Rogers and other specialists like her, enthusiastically following out this practice earnestly.

I have up to this time not been able to cure pyorrhea and make it stay cured, but now, from what I have seen with my own eyes, and what I have been able to accomplish with my hands, I believe for a certainty that pyorrhea can be cured, and made to stay cured by "oral prophylaxis treatment".

The regular and intelligent treatment of the soft tissues of the mouth contiguous with the teeth themselves, after the thorough scaling of the teeth and removal of all deposits, is absolutely essential and unless this thorough cleansing and polishing process is kept up regularly afterwards, the pericemental diseases will recur. There is no doubt of this in my mind. We have some practitioners who claim to cure pyorrhea at once sitting so that it will stay cured. We had a clinic of this kind here a year ago, and I do not know the result of that case, but I do not hear that anyone was so impressed that he has adopted the treatment. I do not believe that we can get permanent results from that kind of treatment. We have a gentlemen in this city whom Dr. Good treated two years ago, by this method, if that tooth has stayed cured so far I shall be very much surprised, because I not do believe the gentlemen has had it treated since. I think that no man can take up this prophylaxis work and follow it out to a successful issue unless he puts himself under the treatment previously, at the hands of an expert such as Dr. Rogers, or Dr. Spalding, who has done this community of dentists as much or more good than any one I know of, because of his earnest efforts to introduce this prophylaxis treatment to the profession and people of our city.

Dr. Spalding spent weeks in giving oral prophylaxis treatment to several of our mouths prior to giving his paper before our State Society last summer, and I was one of his patients. But for that treatment I do not think I should be practicing oral phropylaxis today with any degree of sucess at all, and I believe I am now with some success at least, I attribute much to the fact that Dr. Spalding was persistent in giving me that thorough treatment, which characterizes all his work and is so essential that results and benefits may follow. I have not kept it up all the time since, but I know what the results have been I will say again that if any man thinks of taking up this line of work,he should by all means go and have Dr. Spalding or Dr. Rogers, or some one who is capable of doing the work thoroughly, give them the treatment, then they will know all about it, because I believe they have sifted it to the bottom.

I used to speak to my patients of constitutional treatment just as Dr. Graham does. I now tell them at once that I know that the regular prophylaxis treatment, after a thorough removal of this irritating deposits will cure the disease. I used to recommend some constitutional treatment, but I do not any more. I am not capable of diagnosing the rare cases which need that kind of treatment, so I do not recommend it at all. I have never recommended it in any case where I could see any direct or decided benefits .

The soft deposits about the teeth in treatment of pyorrhea are most important factors. After the first thorough scaling of the teeth and roots, the important thing is to keep the soft deposits off the teeth. I want to refer to one case to verify the importance of this line of work. I had a doctor bring a patient into the office who was highly nervous, she could not bear to have the doctor touch her, but finally I made an appointment with her and she came, and I looked the mouth over, and found she had one or two cavities. I

found the gums pink, red and purple and in a bad condition from deposits. I told the doctor that I believed her nervousness and a neuralgia which kept her awake every night was caused entirely from those deposits. I am not speaking to laud my work in any sense of the word, but this case came to my notice one day, and I gave it one treatment of half an hour, removeing the worse deposits to give her some relief at once. The husband came in the next day and wanted to know if I really believed that treatment was of any value; at the same time he told me that his wife slept all night perfectly easy, and she had not done so before for a week or more. I think that these deposits cause all sorts of troubles that we too often attribute to other causes.

DR. A. A. LAUPPE: I am sure I have nothing to say, except to compliment the assayist on the paper, as the subject is rather a large one. It is peculiar in the way it is put to the profession at the present time and the way it is agitated. I like to read the articles in the various journals, but they are so full of laudation and condemnation that one almost gets disgusted in reading them.

It is peculiar also for the many statements made which seemingly bear no logical reasons, and for the many claims made which have no apparent foundation. I will take up a few of the peculiarities of the propaganda.

For instance, the first thing is the definition. Now prophylaxis means to guard against. It is a noun. Prophylactic, is the agent or measure for guarding against, and is an adjective. Dr. D. D. Smith, says that the two words have separate and distinct meanings. How can the adjective mean something entirely different from the noun. How can a noun be used as an adjective without undergoing some change in its ending, I do not know. Prophylaxis, furthermore, is not defined in any dictionary as an adjective. Take for instance the prophylactic tooth brush. It should be then termed the Prophylaxis tooth-brush.

They tell us the wonders of that hand method and that you cannot thoroughly clean a set of teeth except by the hand method exclusively.

Now I will venture this statement, that you cannot successfully polish a set of teeth by the hand method exclusively. Now if it takes you three hours, or more, to thoroughly polish an unclean set of teeth by the hand method exclusively, and one hour by the engine and hand method, which method would you term the successful method from a practical standpoint?

They also tell us of the wonders of the little wood point. It is a wonder that humanity has any teeth left at all, after reading the articles. They tell us with that little wood point you can get to every part of the teeth. I don't see how you can get to that part of the teeth where caries most readily commences, just at the proximal contact point. When you come to an approximal surface of the teeth there is a triangular space which is not touched at all. When you work the wood point sideways it strikes a tangent, and there is another triangular space which is not struck. You go around a molar or bicuspid, your wood point is wedge shaped and it strikes another tangent. You know as well as I do that you are often obliged to resort to strips charged with pumice and strings charged with pumice to polish the approximal surfaces of the teeth, in spite of that wood point.

They tell us also that the circulation in the enamel is increased. There is no doubt about it that the set of teeth which is polished every month looks fine. The gums are are in a fine condition, the surroundings of it are fine and there is a nice refraction of light there. But let us suppose that you have a dirty face; you wash it and a good color results. It is necessary to say that the blood circulation is increased as a result; or, in regard to the finger nails, when they are manicured, is it necessary to say that the circulation has increased because they look better?

They tell us it's useless to clean the tongue, and yet at the same time they spend hours of time in discussing the cleaning of the teeth. Why neglect the tongue and thoroughly clean the teeth? The tongue is coated often, with dead epithelium and any amount of fermentable material, and why is it useless? It seems to me that it is necessary.

They also tell us of the amount of infection in the mouth and the virulent bacteria, then, why is it that wounds in the mouth will heal more readily than in any other part of the body? Why is there not more auto-infection?

Then they place great stress upon the mucus accumulations and tartar about the teeth as causing decay, and yet it is not a fact. Why is it that teeth like the lower anterior teeth, which are continually bathed in saliva are the last teeth in the mouth to decay?

They bring this subject up as something entirely new, and yet, after all, when you went to college were you not taught all about the serumal calculus and different calculae and that to thoroughly clean a set of teeth you had to do all, this, and if you did not do it after you graduated, who have you to blame? It seems that there is nobody to blame but yourself. The teachings were all right.

They tell us that oral prophylaxis is not "cleaning" teeth. They make the teeth clean, though, while it may not be policy to use the word "cleaning" teeth, yet you do clean the teeth.

In order to get success with this treatment, your patient must be extremely attentive to the daily care of his teeth. Patients must not only brush their teeth before retiring at night, but before and after each meal using an antiseptic tooth wash, tooth powder, quill, etc., Now suppose you have a case which you have put in a healthy condition, and that mouth, like the average persons mouth, is not affected with tartar to any extent, is the monthly treatment, provided your patient will do all of that, an essential, or is it a great extreme? Does the success of this treatment depend so much what the dentsit does as it does on what the patient does? I prefer to be more conservative. You understand the monthly treatment is beyond the means of the average person. The average practitioner cannot apply it in his daily practice. If your patient will do all that is required of him for the success of the treatment, and will use pumice and brush once in two weeks or once a month, I believe they can do as much, without coming every month, as the dentist can, providing they are not troubled with tartar.

It is impracticable to practice daily what Dr. Smith requires. Those thing look pretty in print, but when it comes to applying them it is a different proposition entirely.

The effect of much of this agitation that is going on, it seems to me, is to stimulate to better service along the line of cleaning teeth. We all cannot succeed in it and there will not be much general success if you cannot stimulate some means of a better fee. I divide the cleaning of teeth into two operations, that of scaling the teeth and that of polishing them. I charge a fee for each and I charge accordingly.

When the patients presents themselves to me and say they want their teeth cleaned, I look at them and say that they want to be scaled. Then I tell them that they need polishing. You speak prophylaxis to the average person and they will say, what are you giving me? The young man when he graduated from college tried to do what was right, but when he got out into practice he met the patients of the older practitioners and he found that the older men were cleaning teeth in half an hour, and he had to meet the other mens terms. He did not want to starve, so what was the result. He fell into the same rut that the old men were in and he helped to make it deeper. Now if you do not thoroughly scale the teeth you will have pyorrhea, but if you charge for scaling thoroughly and not treating the gums, there will be less pyorrhea in the future. Pyorrhea is a common disease, and it is not difficult to cure in many instances. With some, however, you cannot cure it.

It is all right to have a high ideal, but you have got to apply all that is going on in your daily practice. Nintynine per cent of dentsits are not practicing this prophylaxis in daily work.

Dr. W. H. Graham: In regard to a tooth that was treated by Dr. Good, of Chicago, I wish to say that it is now nearly two years and it is perfectly cured. I only had the one treatment, about two hours spent on the tooth.

Dr. G. P. Rogers: Dr. Graham suggested that I implied that this was a new thing. The idea I tried to convey in the beginning of my paper was that more than half a century ago men began work along the lines of oral hygiene; and up until the time that Dr. Smith started his prophylaxis treatment and carried it out successfully, there were men working all the time, and that if it had not been for the work these men did, and for the successes of these few men, I do not believe it would have been possible for prophylaxis to have been put on such a firm basis as it is.

Dr. Graham made the remark that he believed that teeth that were in bad condition could be put in a fairly good condition. They can be put into perfect condition, as all those who have been in Dr. Smith's office can testify, unless the peridental supporting tissues are wholly lost.

As to the cause of pyorrhea; why is it that local treatment only will cure this disease, and why if the teeth are kept in perfect condition will this disease not recur; then why with teeth that are kept under regular constitutional treatment will we not get cures?

Dr. Wood's suggestion that every dentist ought to put himself under regular treatment is a very good one, because there are no mouths that are more badly neglected than the mouths of the dentist. I have had occasion to examine the mouths of a number of dentists within the last six months (I think seven), and out of that seven there were four who had gutta percha fillings in several of their teeth.

Dr. Lauppe's remarks were rather lengthy and he brought up a number of points that should be answered. I am afraid, however, there were too many to answer.

As to the term prophylaxis being used as an adjective. He says that it is noun and cannot be used as an adjective. Dr. Smith is the man who first put this treatment on a systematic basis and he ought to have the right to coin a new word when necessary to convey exact ideas. If it is necessary to convert a noun into an adjective he may, to mark the distinction he is obliged to convey. A prophylactic treatment means the prevention of the disease through medicinal means and convey only the idea of quality or degree.

In regard to using the wood point: Dr. Smith used the wood point entirely, but that is not a sign that we should all use it, If we cannot reach the proximal surfaces with the stick of orange wood, use some other thing that will reach it; silk floss is very good for that purpose. If the points get dull you can sharpen them easily.

In regard to circulation in the enamel. There may be no circulation in the enamel, but Dr. Smith can show you case after case where the white spots have disappeared, and these usually do not disappear unless the tooth has been under this treatment.

Another question was that of neglecting the tongue, the care of it. That is another thing Dr. Smith does do. He advocates very strongly that we take care of the surface of the tongue and he cleanses it as much as it is possible to do so.

I think the reason why the lower anterior teeth do not decay is that they are often protected by tartar. In college we were taught to remove this tartar of course, because the mouths were in an unhygiene and unclean condition; we were not given cases of pyorrhea but simple cases of scaling which have caused local irritation.

As for the use of the term "cleaning teeth"; in prophylaxis treatment we do not clean teeth. The patient does that for himself, and we should only do it for him where he cannot do it for himself. After our first treatment, and after he has learned to care for his own mouth, the patient that is doing what he should do, and is caring for himself as he should do, cannot detect any soft accumulation at all. Oftentimes I have seen cases where the gums looked quite angry and there was no evident cause until I started to polish them, and the surfaces then were very slippery, and I am positive that the condition was due simply to these irritating coatings

He says the average practitioner cannot carry out this treatment. The majority do not try to carry it out. There are a great many patients in this city that are leaving their own dentists and going where they can get prophylaxis treatment, simply because their own dentists will not give it to them, or say they do not need it, but the patient wants it, and every patient who has it says that they would not get along without it any more than they would get along without washing their faces.

He says that patients, if you tell them they need prophylaxis treatment, will not know what we are talking about. It is our duty to teach them what it is and what it means, to give them enough treatments to put their mouths in a healthy condition, and then they will know what it means; but we should not use the term if we are not going to practice prophylaxis.

I would like to suggest to every dentist here that he put himself under treatment somewhere, or exchange treatment with some other dentist, so that we may know what we are talking about.

